# Recurrent stroke risk is high after a single cerebrovascular event in patients with symptomatic 50-99% carotid stenosis: a cohort study

**DOI:** 10.1186/1471-2377-14-23

**Published:** 2014-02-04

**Authors:** Elias Johansson, Per Wester

**Affiliations:** 1 Department of Pharmacology and Clinical Neuroscience and Department of Public Health and Clinical Medicine, Umeå University, 901 82 Umeå, Sweden; 2 Department of Public Health and Clinical Medicine, Umeå University, Umeå, Sweden

**Keywords:** Stroke, Carotid stenosis, Risk

## Abstract

**Background:**

Recurrent TIAs are thought to signal a high stroke risk. The aim of this study is to examine if repeated ischemic events increase the risk of recurrent ipsilateral stroke among patients with symptomatic 50-99% carotid stenosis.

**Methods:**

This is a secondary analysis of the ANSYSCAP study, where we analyzed recurrent ipsilateral ischemic stroke before carotid endarterectomy in 230 consecutive patients with symptomatic 50-99% carotid stenosis. Here, we further analyzed the patients according to if they were clinically stable, unstable or highly unstable – respectively defined as having 0, 1 or ≥2 additional ipsilateral events within 7 days before and/or after the ischemic cerebrovascular event for which the patient sought health care (the presenting event).

**Results:**

Of the 230 included patients, 155 (67%) were clinically stable, 47 (20%) were clinically unstable and 28 (12%) were clinically highly unstable. Eighteen patients suffered a stroke within 7 days; of these patients, 12 (67%) were clinically stable. The risk of recurrent ipsilateral ischemic stroke within 7 days was equally high for clinically stable (8%), unstable (9%) and highly unstable (7%) patients. Fourteen patients had 3–11 additional ipsilateral events; of these patients, only one suffered a recurrent ipsilateral ischemic stroke.

**Conclusions:**

The seemingly clinical stable symptomatic 50-99% carotid stenosis patients without additional ipsilateral events have a high risk of recurrent stroke. Patients without additional events should undergo preoperative evaluation and carotid endarterectomy in the same expedient manner as patients with additional events.

## Background

Patients with additional TIAs before a presenting event have a higher risk of recurrent stroke afterwards than patients who seek health care after a single event [1]. For patients experiencing a TIA, the presence of additional events within 7 days before a presenting event adds to the risk stratification after adjustment for the ABCD2 score [1]. The ABCD2 score was amended to ABCD3 by including one or more additional TIAs within 7 days as a risk factor [1]. These findings are based on patients with TIAs of mixed etiologies [1].

Symptomatic 50-99% carotid stenosis incurs a high risk of recurrent stroke [1,2]. Patients with additional ischemic events and symptomatic carotid stenosis have a high risk of perioperative stroke [3-5]. This increased risk may be the lesser of two evils if these patients also have a high risk of recurrent stroke before carotid endarterectomy (CEA). However, previous studies of patients with symptomatic carotid stenosis did not analyze if additional symptoms are risk factors for recurrent stroke before CEA [2,6-8].

The aim of this study was to examine if repeated ischemic events increase the risk of recurrent ipsilateral stroke at 7 and 90 days among patients with symptomatic 50-99% carotid stenosis and otherwise eligible for carotid endarterectomy.

## Methods

This is a secondary subgroup analysis of the Additional Neurological SYmptoms before Surgery of the Carotid Arteries – a Prospective study (ANSYSCAP) [9]. In short, the ANSYSCAP was a single center study that prospectively included 230 consecutive patients with symptomatic 50-99% carotid stenoses. The patients were included at the Umeå Stroke Center in northern Sweden, between 2007-08-01 and 2009-12-31. Many patients (81%) were referred from 11 hospitals. We included patients who were preliminary eligible for CEA, defined as that the patient underwent a specific preoperative evaluation aimed at CEA (n = 226) or would have done so before a major recurrent stroke made CEA not meaningful (n = 4). The selection for this preoperative evaluation was made by two stroke specialists; one in neurology and one in internal medicine together with the vascular surgeon. Co-morbidities and the degree of stroke sequele (in cases with stroke) were taken into account. No specific stroke severity score was used, but as a rule the patient should be relatively cognitive intact (MMSE ≥25) and be able to walk unassisted. The observation period for recurrent ipsilateral ischemic stroke was the first 90 days after the presenting event. If CEA was performed within 90 days, patients were observed up to the surgery. During the observation period, all patients were treated with either anti-platelet or anti-coagulant medication, 93% were treated with blood pressure reducing medication and 90% with lipid-lowering medication. In the primary analysis, we found that TIA or stroke as the presenting event incurred a higher risk of recurrent ipsilateral ischemic stroke than amaurosis fugax, but we detected no differences based on age, sex, or the degree of carotid stenosis on the symptomatic side [9].

In the present analysis, we used the same endpoint as the original study – recurrent ipsilateral ischemic stroke that occurs before CEA and within 90 days after the presenting event. Ipsilateral retinal artery occlusion was also included with this endpoint (henceforth included as “ipsilateral ischemic stroke” at all times except when clearly specified). The endpoints were based on clinical assessment, radiological confirmation was not required. However, brain computer tomography or magnetic resonance tomography was performed to rule out a hemorrhagic or other differential diagnoses. We only analyzed events that occurred before CEA, excluding all perioperative events. The presenting event was defined as the last ischemic cerebrovascular event (stroke, retinal artery occlusion, TIA, or amaurosis fugax) before the patient sought health care. We defined ipsilateral as the same side as the presenting event. Cerebrovascular events were ascertained by a combination of patient interview, medical records from various sources and follow-up by letter and telephone. In the original study, we recorded every previous and future cerebrovascular events over a one year follow-up. To the best of our ability, we recorded the duration and vascular territory (right carotid, left carotid, or posterior circulation) of each event. There were no hemorrhagic events.

We analyzed the patients based on the number of ipsilateral events within 7 days before or after the presenting event. For the analysis of events before the presenting event, we used all ipsilateral events regardless of type (stroke, retinal artery occlusion, TIA, and amaurosis fugax). Since the endpoint was ipsilateral ischemic stroke or ipsilateral retinal artery occlusion after the presenting event, these events were not used when grouping the patients. Therefore, for the analysis of events after the presenting event, we only used ipsilateral TIA and ipsilateral amaurosis fugax (henceforth referred to as ipsilateral events after the presenting event). We only used events that occurred before CEA and before the endpoint, if it was reached.

We grouped the patients based on if they were clinically stable, unstable or highly unstable: Stable patients had no additional events within 7 days before or after the presenting event. Unstable patients had one additional event within 7 days before or after the presenting event. Highly unstable patients had ≥2 additional events within 7 days before and/or after the presenting event.

We compared the prevalence of clinically stable, unstable and highly unstable among the clinical subgroups according to sex, age, degree of symptomatic carotid stenosis, type of presenting event, and TOAST-classification. We analyzed if the delay between the presenting event and CEA was affected by if the patients were clinically stable, unstable or highly unstable. We compared the risk of recurrent ipsilateral ischemic stroke between the clinically stable, unstable and highly unstable patients. We further analyzed if the risk of recurrent ipsilateral ischemic stroke based additional events before the presenting event and after the presenting event separately. Finally, we performed several explorative analyses as specified in the results.

### Ethics

The study was audited by the Umeå research ethics committee, which found that the study did not require committee approval because it was strictly observational. Therefore, informed consent was not obtained, in accordance with the current ethical practice. The study was registered at http://www.clinicaltrials.gov (NCT 00514592) before it was launched. The delay to CEA was not in any way prolonged for the purpose of the study.

### Statistics

We calculated the 90-day risk of recurrent ipsilateral ischemic stroke with Kaplan-Meier curves. We used CEA as a censor; therefore, patients that underwent CEA only contributed to the risk-time before their surgery and all perioperative events were excluded. The risks at 7 days were acquired from this survival analysis. We used the log rank test for differences between subgroups. We also adjusted all findings for age, sex, degree of symptomatic carotid stenosis, and type of presenting event using Cox Regression. The number of days between the presenting event and CEA was non-parametric; therefore, this delay was presented as the median and intra quartile range (IQR). We used the chi-squared-test and Kruskal-Wallis test where appropriate. We used IBM SPSS 20.0 statistical software for all calculations.

## Results

**Figure 1:**
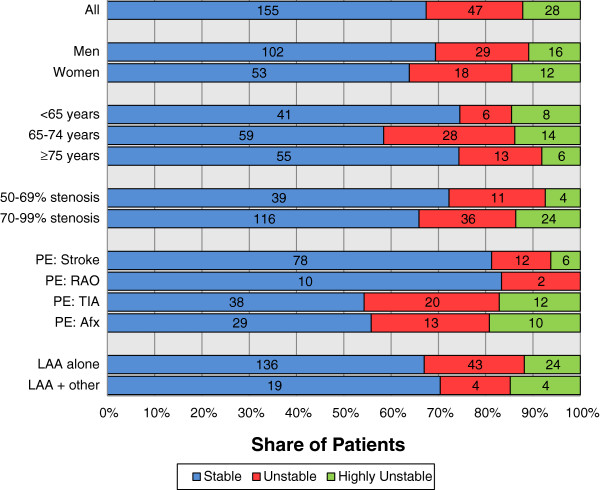
**Prevalence of additional ipsilateral events.** Numbers inside bars denotes number of patients. PE = presenting event; RAO = retinal artery occlusion; Afx = amaurosis fugax; LAA = large artery atherosclerosis

Of the 230 included patients, 155 (67%) were clinically stable, 47 (20%) were clinically unstable and 28 (12%) were clinically highly unstable. Patients with stroke or retinal artery occlusion as presenting event were more often clinically stable than patients with TIA or amaurosis fugax as presenting event (p = 0.004, chi-squared test), see Figure 1. Patients in the intermediate age group (65–74 years) tended to be less often clinically stable than younger (<65 years) or older (≥75 years) patients (p = 0.060, chi-squared test). The delay between the presenting event and CEA, and hence the observation time, is presented in Table 1.

**Table 1:** Delay between the presenting event and CEA based on clinical stability

	**Clinically stable**	**Clinically unstable**	**Clinically highly unstable**
Number of patients	155	47	28
Delay to CEA median weeks (IQR)	4.4 (2.9-8.1)*	4.0 (2.7-6.4)*	2.1 (1.1-3.7)*
CEA within 7 days n (%)	1 (1)	3 (6)	6 (21)
CEA within 14 days n (%)	18 (12)	6 (13)	12 (43)
CEA within 30 days n (%)	56 (36)	22 (47)	20 (71)
CEA within 90 days n (%)	102 (66)	35 (75)	25 (89)

CEA Carotid EndArterectomy. IQR Inter Quartile Range.

* p < 0.001 (Kruskal-Wallis test).

### Additional events and risk of recurrent ipsilateral ischemic stroke

**Figure 2:**
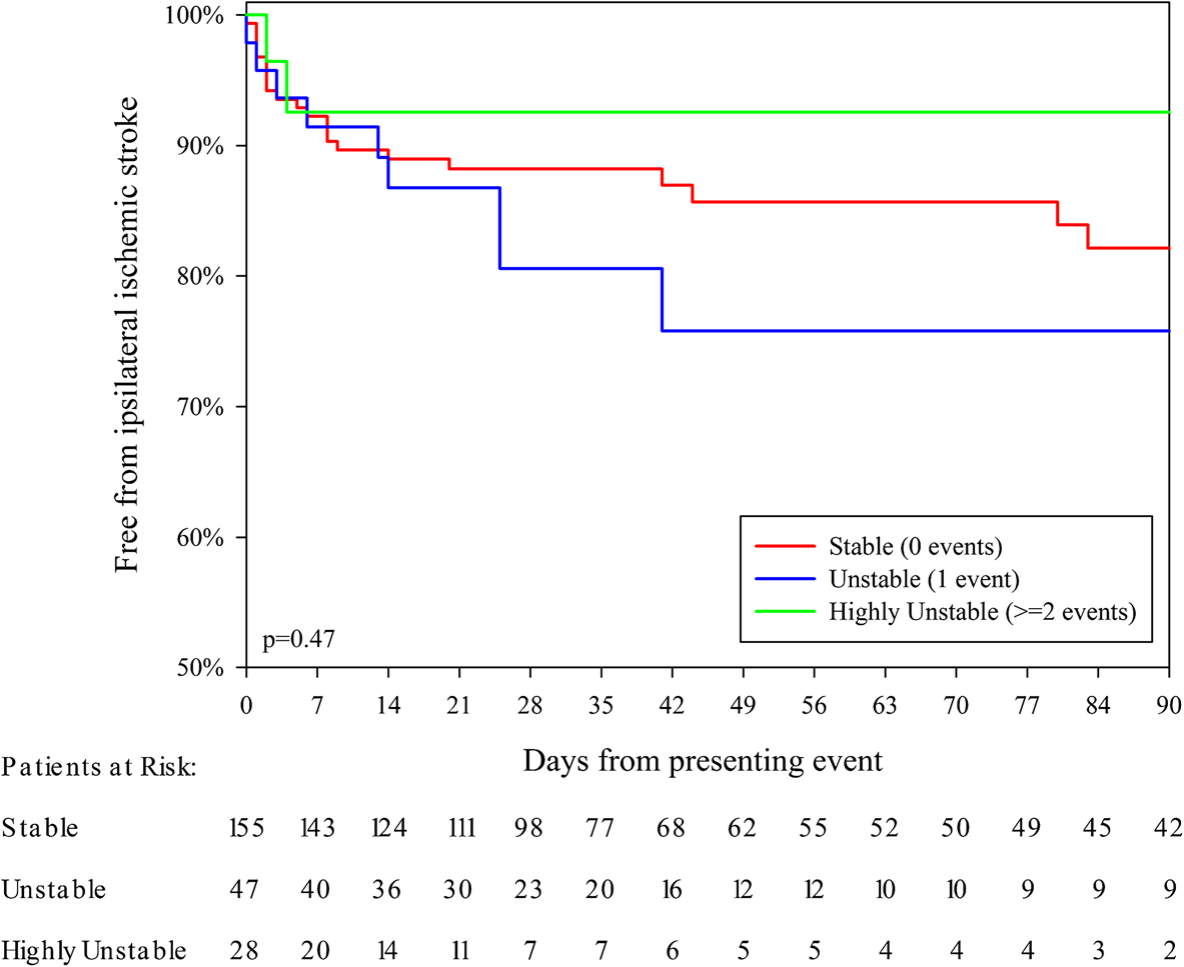
**Kaplan Meier analysis of the risk of recurrent ipsilateral stroke after the presenting event with patients divided by number of events within 7 days before and/or after the presenting event: Clinically stable (0 events), clinically unstable (1 event), and clinically highly unstable (≥2 events).** CEA was used a censor.

**Figure 3:**
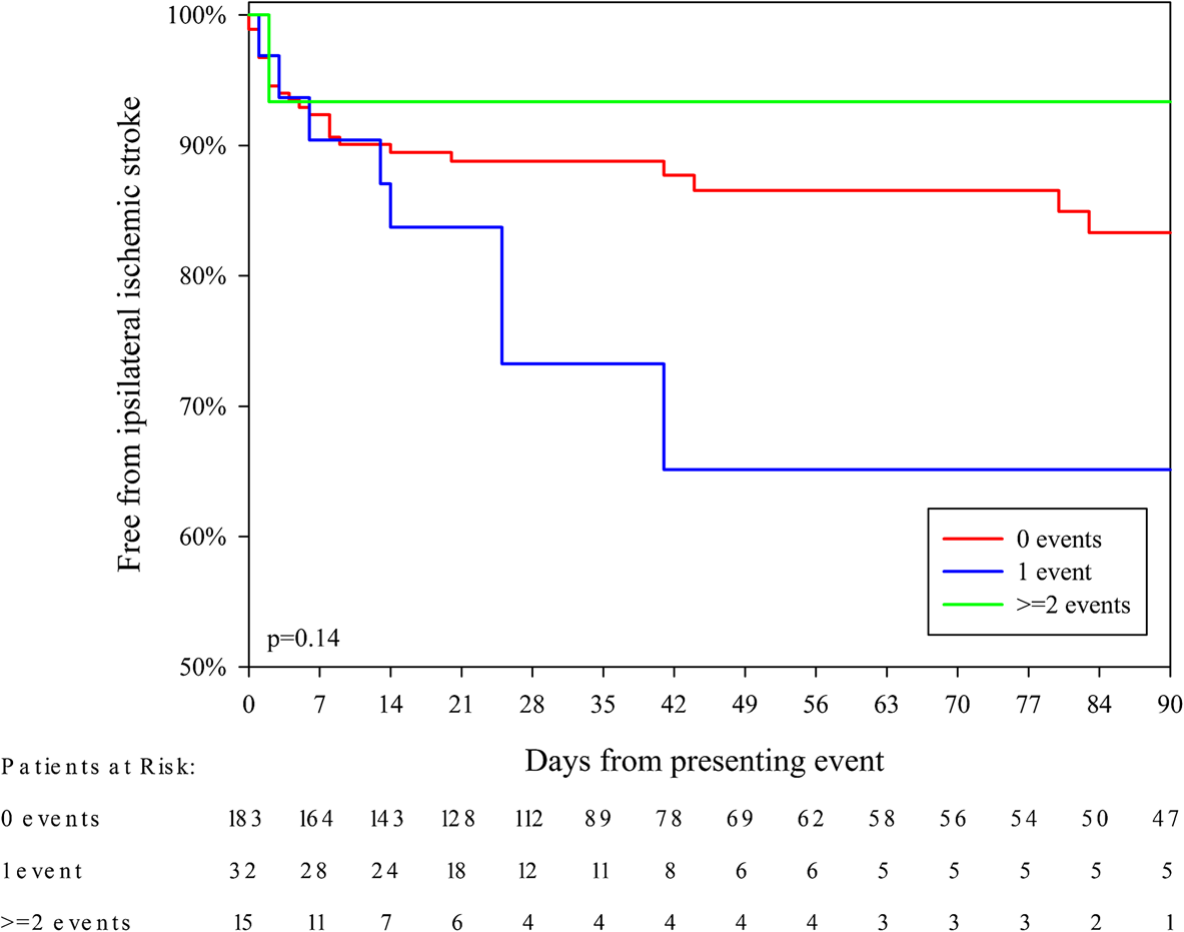
**Kaplan Meier analysis of the risk of recurrent ipsilateral stroke after the presenting event with patients divided by number of additional events within 7 days before the presenting event.** CEA was used a censor.

**Figure 4:**
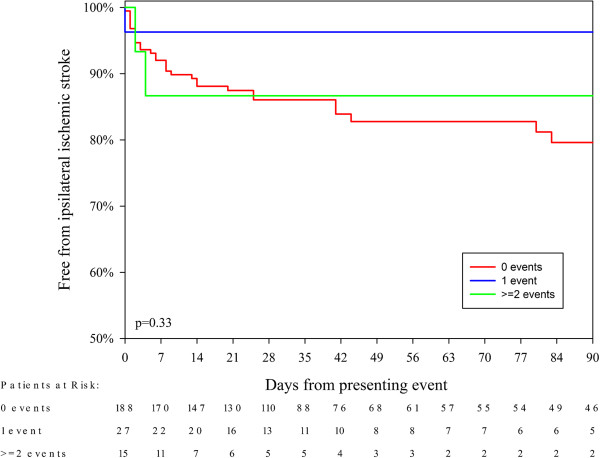
**Kaplan Meier analysis of the risk of recurrent ipsilateral stroke after the presenting event with patients divided by number of additional events within 7 days after the presenting event.** CEA was used a censor.

Thirty-two patients suffered a none-fatal and one patient suffered a fatal recurrent ipsilateral ischemic stroke within 90 days of the presenting event. Eighteen patients suffered a recurrent ipsilateral ischemic stroke within 7 days after the presenting event. Twelve (67%) of these patients were clinically stable, 4 (22%) were clinically unstable, and 2 (11%) were clinically highly unstable, see Table 2. The risk of ipsilateral ischemic stroke was not statistically significantly affected by if the patient was clinically stable, unstable or highly unstable (Table 2, Figure 2). We analyzed the risk of recurrent ipsilateral ischemic stroke based additional events before the presenting event and after the presenting event separately: we found non-significant trends that 1 additional ipsilateral event within 7 days before the presenting event incurred a higher risk of recurrent ipsilateral ischemic stroke than 0 or ≥2 events (Figure 3, Table 2); whereas the opposite pattern was observed in patients with 1 additional event after the presenting event (Figure 4, Table 2). After adjustment for age, sex, degree of symptomatic carotid stenosis and type of presenting event using Cox Regression, all tendencies were weaker or similar, see Table 3.

**Table 2:** Number and risk of ipsilateral ischemic strokes on days 7 and 90 after the presenting event, according to the number of additional events

	**Patients**	**7 days**		**90 days**		**p-value**
		**Strokes**	**Risk**	**Strokes**	**Risk**	
	**n (%)**	n (%*)	**% (95%CI)**	n (%*)	**% (95%CI)**	
Clinically stable	155 (67)	12 (67)	8% (4–12)	22 (67)	18% (10–25)	p = 0.47†
Clinically unstable	47 (20)	4 (22)	9% (1–17)	9 (27)	24% (10–39)	
Clinically highly unstable	28 (12)	2 (11)	7% (0–17)	2 (6)	7% (0–17)	
0 events 7 days before the presenting event	183 (80)	14 (78)	8% (4–12)	24 (73)	17% (10–24)	p = 0.14†
1 event 7 days before the presenting event	32 (14)	3 (17)	10% (0–20)	8 (24)	35% (13–57)	
≥2 events 7 days before the presenting event	15 (7)	1 (6)	7% (0–19)	1 (3)	7% (0–19)	
0 events 7 days after‡ the presenting event	188 (82)	15 (83)	8% (4–12)	30 (91)	20% (13–28)	p = 0.33†
1 event 7 days after‡ the presenting event	27 (12)	1 (6)	4% (0–11)	1 (3)	4% (0–11)	
≥2 events 7 days after‡ the presenting event	15 (7)	2 (11)	13% (0–31)	2 (6)	13% (0–31)	

* Percent of all strokes. † Log rank test. ‡ Events after the presenting event: Only additional TIA or amaurosis fugax that occurred before CEA and before recurrent ipsilateral ischemic stroke were analyzed.

Risk figures derived from Kaplan-Meier analyses.

**Table 3:** Cox regression analyses of the risk of recurrent ipsilateral ischemic stroke

	**Crude hazard ratio**	**p-value**	**Adjusted hazard ratio†**	**p-value**
Clinically stable	1.0	-	1.0	-
Clinically unstable	1.5	p = 0.34	1.3	p = 0.52
Clinically highly unstable	0.6	p = 0.55	0.7	p = 0.58
0 events 7 days before the presenting event	1.0	-	1.0	-
1 event 7 days before the presenting event	2.1	p = 0.07	1.9	p = 0.16
≥2 events 7 days before the presenting event	0.6	p = 0.64	0.9	p = 0.93
0 events 7 days after* the presenting event	1.0	-	1.0	-
1 event 7 days after* the presenting event	0.3	p = 0.17	0.2	p = 0.15
≥2 events 7 days after* the presenting event	1.0	p = 0.97	0.8	p = 0.78

* For events after the presenting event: Only additional TIA or amaurosis fugax that occurred before CEA and before recurrent ipsilateral ischemic stroke were analyzed.

† Adjusted for age, sex, degree of symptomatic carotid stenosis and type of presenting event.

**Figure 5:**
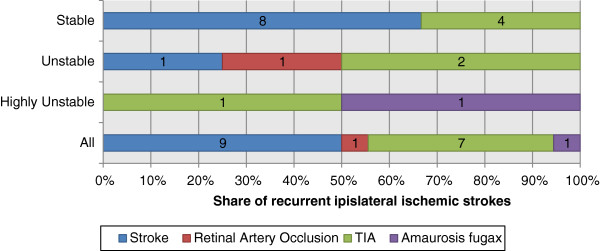
**Type of presenting event among the patients with recurrent ipsilateral ischemic stroke within 7 days divided between clinically stable, unstable and highly unstable.** Numbers inside bars denotes number of patients.

We also conducted explorative analyses: We explored the association between type of presenting event and clinical stability regarding the 18 recurrent ipsilateral ischemic strokes that occurred within 7 days of the presenting event, see Figure 5. We found no interaction for the risk of recurrent ipsilateral ischemic stroke for clinical stability and type of presenting event (p = 0.76; Cox Regression). We further repeated the Kaplan Meier analysis of the risk of recurrent ipsilateral ischemic stroke based on additional events within 7 days before the presenting event, but limited to the 70 patients with TIA (excluding amaurosis fugax) as the presenting event. The findings were similar to those for the entire study with a tendency that 1 additional event before the presenting event incurred a higher risk of recurrent ipsilateral ischemic stroke than 0 or ≥2 events (p = 0.08; log rank test). We also repeated the Kaplan Meier analyses for all patients, but grouped the patients based on if additional ipsilateral events occurred within 90 days (not 7 days) before and/or after the presenting event. All associations were similar or weaker compared to the original analyses.

### Patients with several additional events

Fourteen patients (6%) had ≥3 additional ipsilateral events within 7 days before and/or after the presenting event. Of these patients, 7 (50%) had 3–6 additional ipsilateral events and 7 (50%) had 7–11 additional ipsilateral events. Including the presenting event, 5 (36%) had only amaurosis fugax events, 4 (29%) had only TIA events, and 5 (36%) had a mix of stroke, TIA, and amaurosis fugax events. One (7%) of these patients suffered a recurrent ipsilateral ischemic stroke two days after the presenting event. Eight patients (3%) had ≥10 (range 12–30) additional ipsilateral events within 90 days before and/or after the presenting event. Including the presenting event, 3 (38%) had only amaurosis fugax events, 2 (25%) had only TIA events, and 3 (38%) had a mix of stroke, TIA, and amaurosis fugax events. None of these eight patients suffered a recurrent ipsilateral ischemic stroke after the presenting event.

### Patients with only cerebral events or retinal events

We analyzed how often patients suffer only cerebral events (stroke and TIA) or only retinal events (retinal artery occlusion and amaurosis fugax). First, we considered the 110 patients with additional ipsilateral events (including endpoint events) within 90 days before and/or after the presenting event. In 62/70 (89%) patients with a cerebral presenting event, all the additional events were cerebral. In 31/40 (78%) patients with a retinal presenting event, all the additional events were retinal. Then, we further analyzed the 33 patients that reached the endpoint. In 29/30 (97%) patients with ipsilateral ischemic stroke as the endpoint event, all preceding events were cerebral. In 1/3 (33%) patients with recurrent ipsilateral retinal artery occlusion as the endpoint event, all preceding events were retinal; while, one patient had a single TIA and one had several TIAs as preceding events.

## Discussion

The main findings of this study were that the risk of recurrent ipsilateral ischemic stroke was similar regardless of additional ipsilateral events within 7 days before and/or after the presenting event and that the majority of recurrent strokes occurred in patients without additional events (i.e. in clinically stable patients).

At least in our clinical practice, a patient with additional events is perceived as being at high risk for a recurrent stroke. Therefore, additional events initiate an extraordinary effort with expedient preoperative evaluation and early CEA - hence, the shorter delay to CEA for patients with additional events in this study. During the time period of this study, this meant the patient was transferred the next office day and stayed until the operation. Those measures are currently standard practice; whereas, extraordinary measures are now transfers and operations during evenings and weekends.

One additional event within 7 days before the presenting event had a tendency to incur an increased risk of recurrent stroke, especially among patients with TIA as the presenting event. There are several study limitations to consider when interpreting this finding: (1) Although the patients were prospectively included, the data for events the days before the presenting event were ascertained retrospectively. (2) Patients with additional events underwent CEA early because this reduced the observation time in this group. (3) The most important limitation is the moderate statistical power (230 patients and 33 outcome events), especially when interpreting negative subgroup findings. Given these limitations, it could be a false negative finding that we could not detect any difference in risk between those with and without additional events. However, there was no dose–response relationship (patients with ≥2 additional events seemed to have a lower risk) and no consistency (one additional event after the presenting event tended to be a protective factor rather than a risk factor), which indicate it was not a false negative finding. The lack of dose–response relationship was supported by the finding that patients with several additional events (≥3 within 7 days or ≥10 within 90 days) seemed to continue to have additional transient events, but did not have an increased risk of stroke.

In previous studies of patients with TIAs of mixed etiologies, one or more recurrent event within 7 days before the presenting event was associated with symptomatic 50-99% carotid stenosis and lead to an increased risk of stroke [1]. It is possible that, among patients with TIAs of mixed etiologies, additional events act as risk factors by indicating a high risk etiology (i.e. symptomatic 50-99% carotid stenosis). However, among the patients with this high risk etiology, additional events at presentation are not a risk factor for stroke. This contention has already been reported for large-artery atherosclerosis in a study with 388 patients with TIAs of mixed etiologies [10]. They found large-artery atherosclerosis was the etiology with the highest risk and additional events at presentation were more common (p < 0.001) among patients with large-artery atherosclerosis (42%) than patients with cardioembolism (17%), small vessel disease (13%), other causes (6%), and undetermined causes (24%). Among patients with large-artery atherosclerosis, 16% of patients with additional events at presentation suffered a stroke during the 90 days follow-up, compared to 23% of patients without additional events at presentation (p = 0.49). Although an increased risk for patients with additional events cannot be ruled out based on our results, it is clear that patients without additional events have a high risk of stroke recurrence and most of the recurrent stroke occurred in this group. Thus, it was common that patients sought health care for the first event and suffered a stroke within 7 days, without a transient event in between.

There are two separate clinical implications for the findings in this study. (1) The risk of recurrent ipsilateral ischemic stroke was not increased for patients with additional events. This might be a false negative finding and should be confirmed in further studies; although, the clinical implication is that the risk of ipsilateral ischemic stroke in the acute phase might not be sufficiently high to warrant an emergent CEA, which has a very high risk of perioperative events (11-20%) among patients with unstable symptoms [3-5]. Thus, it is possible that initial conservative treatment and delayed CEA (perhaps >48 hours since the last event) are more beneficial for these patients. (2) The risk of recurrent ipsilateral ischemic stroke was high among the patients without additional events and most recurrent strokes occurred in this group. Therefore, these patients should undergo an expedient preoperative evaluation for an early CEA. Thus, there is a need to extend expedient management, which might be reserved for patients with additional events, to patients without additional events – i.e. to all patients.

## Conclusions

Patients with a symptomatic 50-99% carotid stenosis have a high risk of recurrent stroke even if they do not experience additional events. The majority of the recurrent strokes occur among patients without additional events; thus, these patients should undergo preoperative evaluation and carotid endarterectomy in the same expedient manner that currently might be limited to patients with additional events.

## Abbreviations

ANSYSCAP: Additional neurological symptoms before surgery of the carotid arteries – a prospective study; CEA: Carotid endarterectomy; TIA: Transient ischemic attack.

## Competing interests

The authors declare that they have no competing interests.

## Authors’ contributions

EJ Designed the main study, gathered all data, designed the current analysis, conducted the current analysis and wrote the article draft. PW Co-designed the main study, co-designed the current analysis and revised the article. Both authors read and approve the final manuscript.

## Pre-publication history

The pre-publication history for this paper can be accessed here:

http://www.biomedcentral.com/1471-2377/14/23/prepub
